# Upregulation of LRRK2 following traumatic brain injury does not directly phosphorylate Thr^175^ tau

**DOI:** 10.3389/fncel.2023.1272899

**Published:** 2023-11-08

**Authors:** Neil Donison, Matthew Hintermayer, Maegha Subramaniam, Erin Santandrea, Kathryn Volkening, Michael J. Strong

**Affiliations:** ^1^Molecular Medicine Group, Robarts Research Institute, Schulich School of Medicine and Dentistry, Western University, London, ON, Canada; ^2^Department of Clinical Neurological Sciences, Schulich School of Medicine and Dentistry, Western University, London, ON, Canada

**Keywords:** microtubule-associated protein tau, leucine-rich repeat kinase 2, chronic traumatic encephalopathy, traumatic brain injury, amyotrophic lateral sclerosis, tauopathy, phosphorylation

## Abstract

Phosphorylated microtubule-associated protein tau (tau) aggregates are a pathological hallmark of various neurodegenerative diseases, including chronic traumatic encephalopathy and amyotrophic lateral sclerosis with cognitive impairment. While there are many residues phosphorylated on tau, phosphorylation of threonine 175 (pThr^175^ tau) has been shown to initiate fibril formation *in vitro* and is present in pathological tau aggregates *in vivo*. Given this, preventing Thr^175^ tau phosphorylation presents a potential approach to reduce fibril formation; however, the kinase(s) acting on Thr^175^ are not yet fully defined. Using a single controlled cortical impact rodent model of traumatic brain injury (TBI), which rapidly induces Thr^175^ tau phosphorylation, we observed an upregulation and alteration in subcellular localization of leucine-rich repeat kinase 2 (LRRK2), a kinase that has been implicated in tau phosphorylation. LRRK2 upregulation was evident by one-day post-injury and persisted to day 10. The most notable changes were observed in microglia at the site of injury in the cortex. To determine if the appearance of pThr^175^ tau was causally related to the upregulation of LRRK2 expression, we examined the ability of LRRK2 to phosphorylate Thr^175^*in vitro* by co-transfecting 2N4R human WT-tau with either LRRK2-WT, constitutively-active LRRK2-G2019S or inactive LRRK2-3XKD. We found no significant difference in the level of pThr^175^ tau between the overexpression of LRRK2-WT, -G2019S or -3XKD, suggesting LRRK2 does not phosphorylate tau at Thr^175^. Further, downstream events known to follow Thr^175^ phosphorylation and known to be associated with pathological tau fibril formation (pSer^9^-GSK3β and pThr^231^ tau induction) also remained unchanged. We conclude that while LRRK2 expression is altered in TBI, it does not contribute directly to pThr^175^ tau generation.

## 1. Introduction

Traumatic brain injury (TBI) is one of the leading causes of disability worldwide, affecting over 50 million people annually (Dewan et al., 2019; James et al., 2019). It is a known risk factor for neurodegenerative diseases, including chronic traumatic encephalopathy (CTE) (McKee et al., 2009; Stein et al., 2015), amyotrophic lateral sclerosis (ALS) (Chen et al., 2007; Daneshvar et al., 2021) and Alzheimer’s disease (Guo et al., 2000; Fleminger, 2003). The pathophysiology of TBI is a complex and multifaceted cellular process which includes the pathological phosphorylation of the microtubule-associated protein tau. Tau is a multifunctional protein expressed throughout the central nervous system and mainly enriched in the axons of neurons (Binder et al., 1985). The primary function of tau is the assembly and stabilization of microtubules. However, the phosphorylation of specific residues can induce a conformational change that disrupts its ability to bind and stabilize microtubules (Lindwall and Cole, 1984; Noble et al., 2013). The dissociation of tau from microtubules increases its propensity to form soluble monomeric tau species, which can dimerize and aggregate into paired helical filaments and insoluble filamentous fibrils (Lee et al., 1991). Pathologically phosphorylated tau fibrils, either bearing unique phospho-epitopes or evident as an increase in total tau phosphorylation, is a neuropathological hallmark of various neurodegenerative diseases, including CTE (Corsellis et al., 1973; Omalu et al., 2005; McKee et al., 2009; Kanaan et al., 2016), ALS with cognitive impairment (ALSci) (Yang et al., 2003; Strong et al., 2006; Yang and Strong, 2012) and Alzheimer’s disease (Grundke-Iqbal et al., 1986; Goedert et al., 1989; Hasegawa et al., 1992).

We have previously shown that tau phosphorylated Thr^175^ (pThr^175^ tau) is present in many tauopathies, including CTE, ALS, ALSci and Parkinson’s disease (PD) (Strong et al., 2006; Gohar et al., 2009; Moszczynski et al., 2017, 2018). However, it is not observed in fetal brain tissue (Watanabe et al., 1993; Morishima-Kawashima et al., 1995) or healthy aged controls (Moszczynski et al., 2017), suggesting that it is specific to pathologic states. It is also known that pThr^175^ tau can initiate a cellular cascade that leads to tau fibril formation and cell death (Gohar et al., 2009; Moszczynski et al., 2015). We have previously shown both *in vitro* and *in vivo* that the presence of pThr^175^ tau is associated with tau N-terminus phosphatase activating domain (PAD) exposure which leads to glycogen synthase kinase 3β (GSK3β) activation and the subsequent phosphorylation of Thr^231^ (pThr^231^ tau) (Moszczynski et al., 2015; Hintermayer et al., 2019). This final step is associated with pathological tau oligomer formation. Using an experimental model of TBI in rodents (Moszczynski et al., 2018; Hintermayer et al., 2019), we have shown that within the first 10 days post-injury, there is evidence of pThr^175^ tau immunoreactive inclusions, followed by GSK3β activation and pThr^231^ tau-positive fibrils (Hintermayer et al., 2019).

The mechanism by which Thr^175^ tau is phosphorylated is unknown. Amongst the multiple kinases known to phosphorylate tau (Martin et al., 2013), Leucine-rich repeat kinase 2 (LRRK2) has been suggested as being capable of phosphorylating Thr^175^ (Kawakami et al., 2012; Ujiie et al., 2012; Bailey et al., 2013). LRRK2 is a unique multifunctional protein that contains both a C-terminal kinase domain and a Ras of complex (ROC) GTPase domain. Further, missense mutations in *LRRK2* represent a significant cause of PD (Paisán-Ruíz et al., 2004; Zimprich et al., 2004; Monfrini and Di Fonzo, 2017), including the kinase domain mutation variant p.G2019S which increases kinase activity (Gilks et al., 2005; Steger et al., 2016).

The link between LRRK2 activation, tau phosphorylation and a degenerative disease state is supported by the observation that neurofibrillary tangles containing pathologically phosphorylated tau are present in a significant percentage of PD patients with *LRRK2* mutations (Arima et al., 1999; Wills et al., 2010; Henderson et al., 2019). LRRK2 expression is increased in animal models of TBI in which pathological tau fibril formation is a neuropathological hallmark (Bae et al., 2018; Rui et al., 2018). LRRK2 has been shown to phosphorylate tau at Thr^181^, Ser^202^ and Thr^205^ (Kawakami et al., 2012; Ujiie et al., 2012; Bailey et al., 2013). *In vitro*, constitutively active LRRK2 (LRRK2-G2019S), when co-transfected with human tau, is associated with Thr^175^ tau phosphorylation (Bailey et al., 2013). Whether the increased expression of LRRK2 *in vivo* following TBI is explicitly associated with Thr^175^ tau phosphorylation is not known.

Here, we show that in a rodent model of TBI, LRRK2 expression increases at the site of cortical injury and in the hippocampus within the first 10 days post-injury, which temporally aligns with the induction of pThr^175^ tau. However, *in vitro*, using HEK293T cells, we found that LRRK2 is not directly responsible for the phosphorylation of Thr^175^ tau.

## 2. Materials and methods

### 2.1. Animals and controlled cortical impact injury

All experimental protocols were approved by the Western University Animal Care Committee (AUP #2017-135) in accordance with the Canadian Council on Animal Care. The tissues used in these experiments were generated and collected during previous work and can be found detailed in Hintermayer et al. (2019). In short, three-month-old Sprague Dawley (Charles River) rats were subject to a single controlled cortical impact injury (CCI) (3.5 m/s, 2.0 mm depth, 500 ms dwell time). Brain lysates and fixed brain tissue were generated during this previous study.

### 2.2. DAB immunohistochemistry

Three animals from each time point were used for immunohistochemical analysis. Animals were perfused with heparin-saline (0.9% NaCl), fixed with 4% paraformaldehyde and 6 μm sections mounted on glass microscope slides.

For routine immunohistochemistry, antigen retrieval was performed in 10 mM sodium citrate (0.05% Tween 20, pH 6.0) using a pressure cooker. Slides were cooled, washed in PBS, and endogenous peroxidase quenched with 3% hydrogen peroxidase in PBS for 5 min. Slides were then washed in PBS and blocked in 5% bovine serum albumin (BSA) for 1h at RT in 0.3% Triton-X in PBS. Primary antibody incubation against LRRK2 (1:1000, Abcam #133474) was performed at 4°C overnight in blocking solution. Slides were then washed in PBS and incubated with biotin-conjugated Rabbit IgG secondary antibody (1:200, Vectastain Elite ABC Kit Peroxidase #PK-6101) diluted in blocking solution for 1h at RT. As per the manufacturer’s protocol, slides were incubated with the Vectastain ABC reagent for 30 min. The antigen target was visualized with 3,3′-diaminobenzidine (DAB). Counterstaining was performed with Harris’ hematoxylin. Individual images were captured on an Olympus BX45 light microscope (Olympus Life Sciences), and a Leica Aperio AT2 microscope slide scanner (Leica Biosystems) captured tile scan images.

### 2.3. Immunofluorescence microscopy

For immunofluorescence staining, antigen retrieval was conducted with Tris-EDTA buffer (10 mM Tris, 1 mM EDTA, 0.05% Tween 20, pH 9.0) in a pressure cooker. Slides were cooled and washed in PBS, then blocked (blocking solution: 3% BSA, 0.1 M glycine, 0.25% Triton X-100 in PBS) for 1 h at RT. Primary antibodies against LRRK2 (1:500, Abcam #133474), NeuN (1:500, Sigma-Aldrich #MAB377), GFAP (1:200, BD Biosciences #556330) or Iba1 (1:500, Sigma-Aldrich #MABN92) were diluted in blocking solution and incubated overnight at 4°C. Slides were then washed in PBS and incubated with Alexa Fluor-conjugated secondary antibodies: Donkey anti-Rabbit Alexa488 (1:1000, Thermo Fisher Scientific #A21206) or Goat anti-Mouse Alexa633 (1:200, Thermo Fisher Scientific #A21050) in blocking solution for 1 h at RT, washed in PBS, counterstained with Hoechst 33258 (1:1000, Biotium #40045), washed in PBS and coverslipped with Immuno-mount (Fisher Scientific #9990402). Images were captured on a Leica SP8 confocal microscope (Leica Microsystems), and deconvolution post-processing was completed using Fiji (Schindelin et al., 2012).

We quantified the cell-specific (neuronal, microglia, astrocytic) expression of LRRK2 near the site of injury by counting the number of NeuN^+^, Iba1^+^ or GFAP^+^ LRRK2 immunoreactive cells per 180 μm^2^. Only cells with a defined nucleus were included for the quantification. Three representative images for each animal were taken at 63× and averaged. A total of three animals were analyzed per timepoint and condition. For neurons, in addition to the percentage of neurons expressing LRRK2, we described the pattern of cellular somatotopic immunoreactivity.

### 2.4. Plasmid constructs

eGFP-WT 2N4R human tau has been previously described (Gohar et al., 2009). 2XMyc-LRRK2-WT (Addgene plasmid # 25361; http://n2t.net/addgene:25361; RRID:Addgene_25361; WT LRRK2), 2XMyc-LRRK2-G2019S (Addgene plasmid # 25362; http://n2t.net/addgene:25362; RRID:Addgene_25362; G2019S LRRK2), 2XMyc-LRRK2-3XKD (Addgene plasmid # 25366; http://n2t.net/addgene:25366; RRID:Addgene_25366; 3XKD LRRK2) and 2XMyc-LRRK2-K1906M were gifts from Mark Cookson (Greggio et al., 2008; Chia et al., 2014). eGFP-Rab10 was a gift from Marci Scidmore (Addgene plasmid # 49472; http://n2t.net/addgene:49472; RRID:Addgene_49472) (Rzomp et al., 2003).

### 2.5. Cell culture and transfection

HEK293T (ATCC #CRL3216) cells were cultured in Dulbecco’s Modified Eagle Medium (DMEM; Invitrogen) supplemented with 10% fetal bovine serum (FBS; Invitrogen) and 0.5% penicillin-streptomycin (Invitrogen) at 37°C and 5% CO_2_. Cells were transfected with Lipofectamine 2000 (Invitrogen) according to the manufacturer’s protocol. Cells were transiently co-transfected with eGFP-WT 2N4R tau, and 1, 2 or 4 μg of LRRK2-WT, LRRK2-G2019S, LRRK2-3XKD or LRRK2-K1609M to examine LRRK2’s effect on tau phosphorylation. HEK293T cells were also transfected with LRRK2-WT, LRRK2-G2019S, LRRK2-3XKD or LRRK2-K1609M and eGFP-Rab10 to confirm LRRK2 activity. All transient transfections were allowed to express for 48 h.

### 2.6. Protein extraction, western blot and slot blot

At 48 h post-transfection, cells were washed briefly on ice with ice-cold PBS and then lysed using NP40 lysis buffer (50 mM Tris pH8.0, 100 mM sodium chloride, 1 mM EDTA pH 8.0, 1% NP40, 10% glycerol) with Halt^™^ Protease Inhibitor Cocktail (Thermo Fisher Scientific #78438), sonicated and then pre-cleared (10,000 g, 4°C, 20 min). Protein concentration was determined using DC Protein Assay (BioRad #5000112) as per the manufacturer’s protocol.

For Western blots, equal amounts of total protein were mixed with 5× Laemmli buffer and denatured at 95°C for 10 min. Protein separation was performed using 6%–10% SDS-PAGE with subsequent transfer to 0.2 μM nitrocellulose membrane (Bio-Rad #1620112). Membranes were briefly washed with Tris-buffered saline with 0.1% Tween 20 (TBST) and then blocked with 5% BSA in TBST for 1 h. Primary antibodies against LRRK2 (1:1000; Abcam #ab133474), pThr^175^ tau (1:2500; 21st Century #MM-0.147-P), pThr^231^ tau (1:2500; Thermo Fisher #MN1040), GFP (1:2500; Invitrogen #A-11121), pSer^9^ GSK3β (1:1000; Abcam #ab107166), pRab10 (1:1000; Abcam # ab230621), GSK3β (1:5000; BD Biosciences #610202), or GAPDH (1:4000, Abcam #ab9485) were incubated in blocking solution overnight at 4°C. Membranes were washed in TBST and then incubated at RT in HRP-conjugated goat anti-rabbit (1:3000, Invitrogen #65-6120) or goat anti-mouse (1:3000, Bio-Rad #1706516) for 1 h, washed and signals visualized using chemiluminescent substrate (Perkin Elmer #NEL103E001EA) and a Bio-Rad ChemiDoc XRS+ Gel Imaging System (Bio-Rad). Densitometric analysis was conducted using ImageLab software (Bio-Rad). Membranes were stripped using Restore^™^ western blot stripping buffer (Thermo Fisher Scientific #21059) for 20 min at 37°C and 20 min at RT, then washed with TBST and incubated in primary and secondary antibodies as described above for subsequent probing.

For slot blots, the nitrocellulose membrane was pre-soaked in TBS and placed in the Bio-Dot SF Microfiltration Apparatus (Bio-Rad #1706542). The membrane was rehydrated by adding TBS to each well, followed by adding equal amounts of protein diluted to a final volume of 200 μL with lysis buffer. Once the samples had been filtered through the membrane by gentle vacuum, the membrane was rewashed with TBS. The membrane was then incubated in 5% BSA in TBST blocking solution for 1h at room temperature. The primary antibody against LRRK2 (1:1000; Abcam #133474) was incubated overnight at 4°C in blocking solution. Membranes were washed in TBST and incubated with HRP-conjugated goat anti-rabbit secondary antibody (1:3000, Invitrogen #65-6120) for 1h at room temperature. Membranes were washed and visualized as per above with a chemiluminescent substrate.

### 2.7. Statistical analyses

Statistical analyses were conducted using GraphPad Prism 9 (GraphPad Software, California, United States). Data are represented as mean ± SEM. One-way or two-way ANOVA with Tukey’s and Šidák’s multiple comparisons tests were used for statistical analysis. Statistical significance was defined as *p* < 0.05.

## 3. Results

### 3.1. pThr^175^ tau and LRRK2 protein expression is increased following TBI

**Figure 1:**
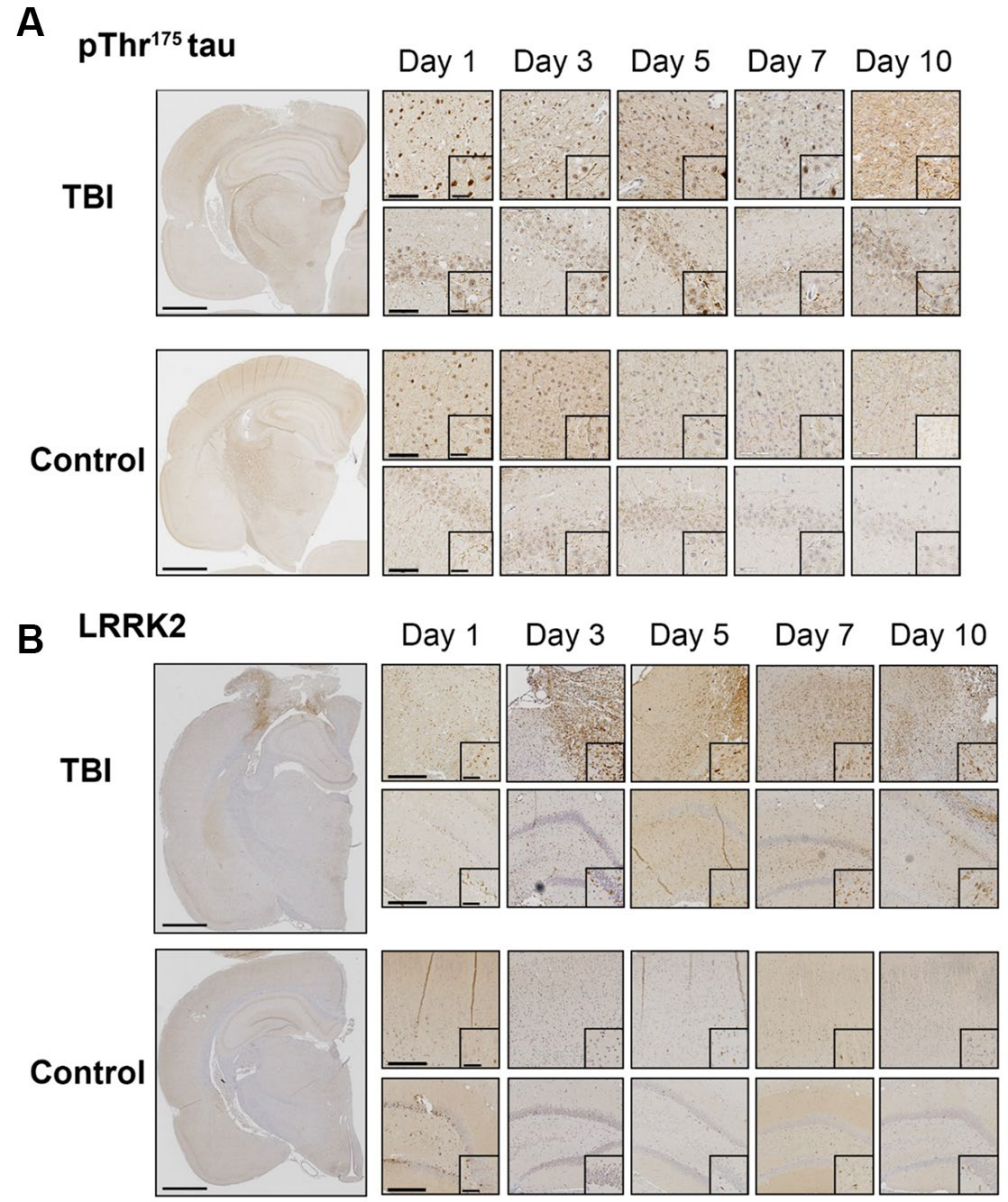
TBI increases pThr^175^ tau and LRRK2 acutely following injury. **(A)** Representative images of pThr^175^ tau in TBI and control animals at days 1, 3, 5, 7 and 10 post-injury. pThr^175^ tau is observed in the cortex (top panel) and hippocampus (bottom panel) of TBI animals as early as one-day post-injury. At day 10, pThr^175^ tau pathology is observed as dense corkscrew neurons and dystrophic neurites in TBI animals. In controls, pThr^175^ tau immunoreactivity is less and does not present as stereotypical tau pathology. **(B)** Representative images of total LRRK2 in TBI and control animals at days 1, 3, 5, 7 and 10 post-injury. Immediately following TBI, LRRK2 is increased primarily in the cortex adjacent to the injury (top panel) and, to a lesser extent, in the hippocampus (bottom panel). LRRK2 is present on day one and increases until day 5, where it remains elevated until day 10. In non-injured controls, total LRRK2 remains low. Compared to controls, LRRK2 expression is mislocalized to the neurites following TBI. Images were taken at 1×, 10× and 40×. Scale bars: 1500 μm (1× images), 300 μm (10× images) and 60 μm (40× images).

We first sought to confirm LRRK2 as a candidate kinase for the phosphorylation of Thr^175^ tau following TBI. Using immunohistochemistry, we examined the temporal and spatial expression of pThr^175^ tau and total LRRK2 in the first 10 days post-injury in rats subject to a single CCI model of TBI. Consistent with our previous data (Hintermayer et al., 2019), pThr^175^ tau was present in TBI rodents one-day post-injury with evidence of pThr^175^ tau immunoreactive fibrils in the cortex, hippocampus, and corpus callosum detected by day 10 (Figure 1A). Stereotypical tau pathology in the form of dystrophic and corkscrew neurites, and neuropil granules was evident. Non-injured controls displayed consistently low levels of pThr^175^ tau (Figure 1A).

Total LRRK2 expression in injured animals was increased in the pericontusional penumbra and hippocampus within the first 10 days compared to non-injured controls (Figure 1B). Specifically, total LRRK2 expression appeared elevated in TBI rodents as early as day 1, peaked at day 5, and persisted to day 10 (Figure 1B). Notably, the greatest DAB intensity (interpreted as the highest level of LRRK2 expression) was detected in cells adjacent to the injury site. This increased expression of LRRK2 was temporally aligned with increased pThr^175^ tau following TBI.

**Figure 2:**
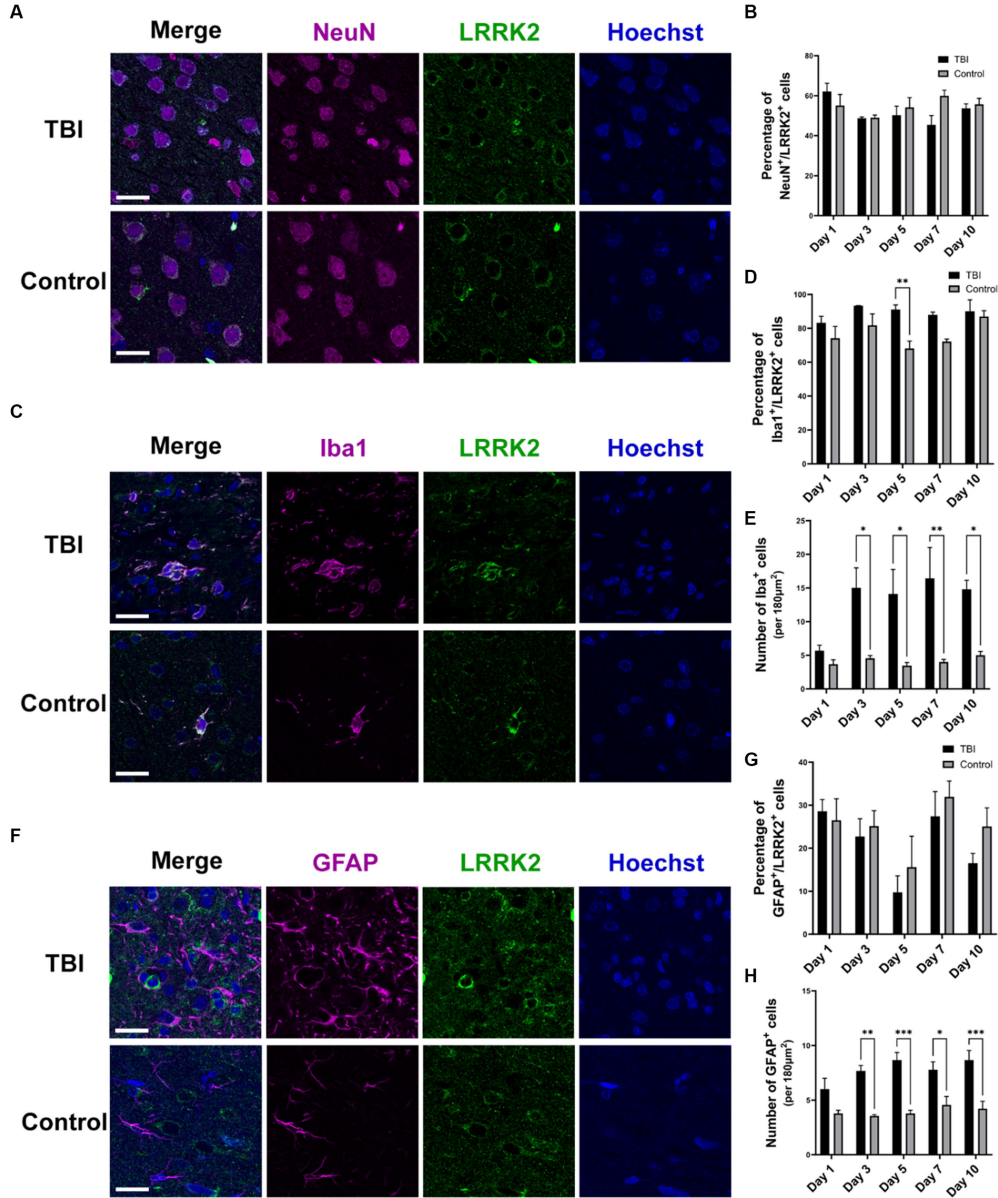
LRRK2 is expressed in neurons, microglia and astrocytes following TBI. **(A)** Immunofluorescence double-labelling of LRRK2 (green) and neurons (NeuN-magenta) in TBI and control animals. **(B)** Quantification of the percent of neurons that express LRRK2. LRRK2 is expressed equally in neurons in TBI (black histogram bars) and control animals (grey histogram bars). **(C)** Immunofluorescence double-labelling of LRRK2 (green) and microglia (Iba1-magenta) in TBI and control animals. LRRK2 was highly co-localized with activated microglia following TBI. **(D)** Quantification of the percent of microglia that express LRRK2. The percentage of microglia that express LRRK2 is equal between TBI (black histogram bars) and controls (grey histogram bars), with the exception of day 5 (*p* = 0.0085). **(E)** Quantification of the total amount of Iba1^+^ microglia per 180μm^2^. Beginning day 3 post-TBI (black histogram bars), the total number of Iba immunoreactive microglia was significantly increased compared to non-injured control animals (grey histogram bars). **(F)** Immunofluorescent double-labelling of LRRK2 (green) and astrocytes (GFAP-magenta) in TBI and control animals. TBI increased reactive astrocytes, which rarely showed co-localization with LRRK2, although GFAP^+^ astrocytes were seen to localize adjacent to LRRK2^+^ cells. **(G)** Quantification of the percent of astrocytes that express LRRK2. LRRK2 is expressed at an equal percentage in GFAP^+^ astrocytes in both TBI and controls. **(H)** Quantification of the total amount of GFAP^+^ astrocytes per 180μm^2^. Similar to the microglial observation, from day 3 onwards, the total number of GFAP^+^ astrocytes was significantly increased in TBI animals (black histogram bars) compared to controls (grey histogram bars). Images were taken at 63×. Scale bars: 20 μm. A two-way ANOVA followed by Šidák’s post-hoc test was conducted. ^*^p < 0.05, ^**^p < 0.005, and ^***^p < 0.0005.

In addition to elevated total levels, we evaluated the cellular pattern of neuronal LRRK2 expression. In non-injured controls, LRRK2 expression was predominantly restricted to the cytosol. In contrast, in TBI animals, we observed that the neuronal localization of LRRK2 appeared to be both perikaryal and neuritic, with more pronounced axonal LRRK2 expression. Despite this, we did not observe an increase in the percentage of LRRK2 immunoreactive neurons between control and TBI animals (Figures 2A,B). These data show that increased LRRK2 expression in TBI rodents spatially aligns with increased pThr^175^ tau, which further supported the rationale to investigate LRRK2 as a candidate kinase for the phosphorylation of tau at Thr^175^.

### 3.2. LRRK2 is increased in microglia and astrocytes

To further understand the relationship between LRRK2 and pThr^175^ tau, and LRRK2 and TBI, we investigated the cellular expression of LRRK2 following TBI. Using immunofluorescence, we examined in which specific cell type LRRK2 expression was increased by double-labelling of LRRK2 and neurons (NeuN), microglia (Iba1), or astrocytes (GFAP). In TBI animals, LRRK2 expression was detected to some extent in all cell types, neurons (Figure 2A), microglia (Figure 2C) and astrocytes (Figure 2F). The most prominent expression of LRRK2 was observed in microglia, with about 80% of Iba1^+^ microglia expressing LRRK2 (Figures 2C,D). The microglia in TBI animals had a stereotypical activated, ameboid morphology associated with inflammation and diseased states. While in general, there was no difference in the percentage of microglia that expressed LRRK2 between TBI and non-injured controls (with the exception of day 5) (Figure 2D), the total number of microglia was significantly increased in TBI compared to controls from day 3 to 10 (Figure 2E). Similarly, while there was no change in the percentage of astrocytes that expressed LRRK2 following TBI (Figure 2G), there was a significant increase in the total number of astrocytes in TBI animals compared to non-injured controls (Figure 2H). The percentage of LRRK2-positive astrocytes was much lower than LRRK2-positive microglia, with only 10%–30% of astrocytes expressing LRRK2. Despite the scarcity of LRRK2 and GFAP co-localization, astrocytes in TBI animals adopted a reactive state and were often observed directly adjacent to LRRK2^+^ cells, with their processes extended. Thus, the increase in total LRRK2 expression in TBI can be attributed to an increase in total number of LRRK2 immunoreactive microglia and astrocytes.

### 3.3. LRRK2 does not phosphorylate Thr^175^ tau in HEK293T cells

**Figure 3:**
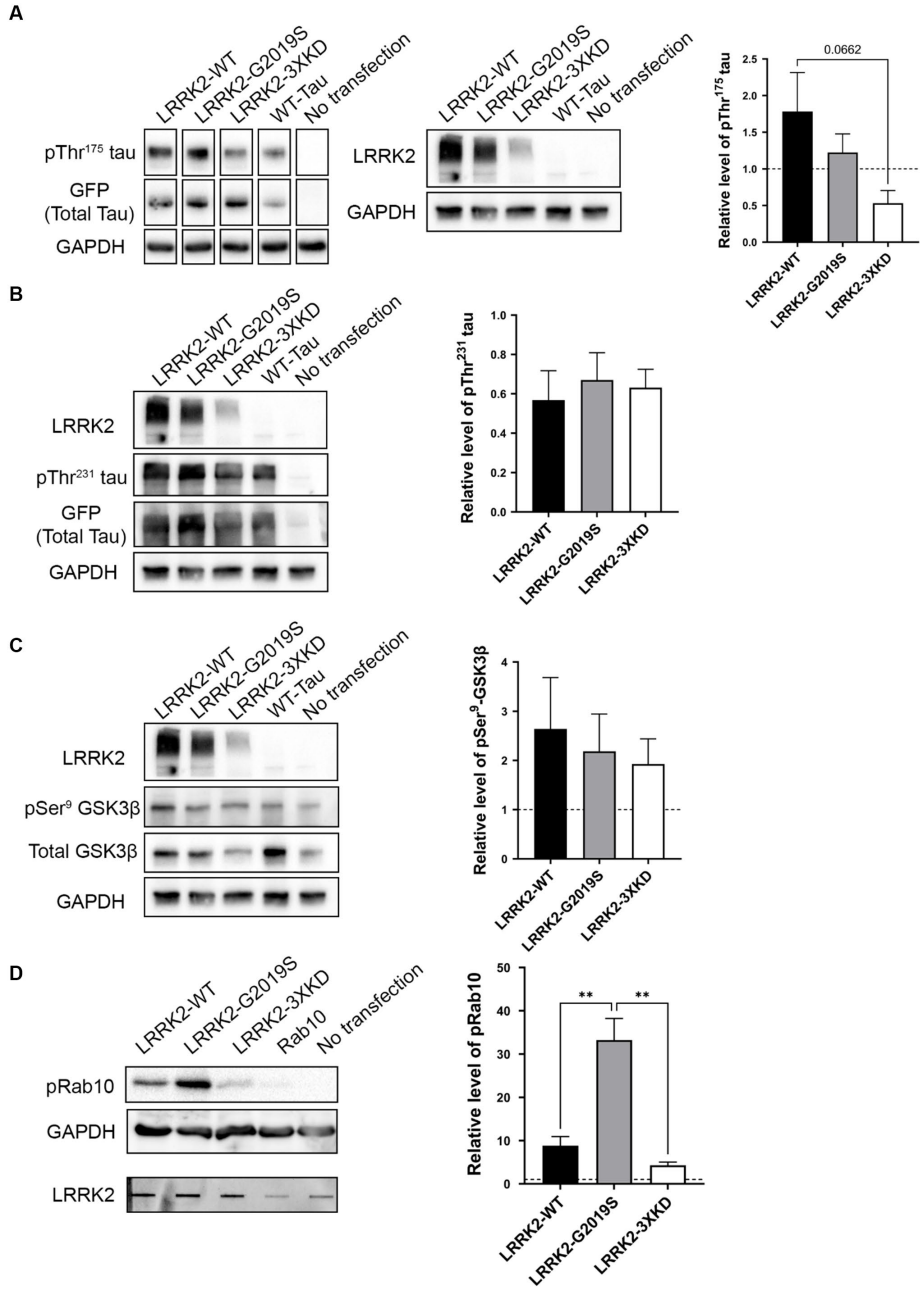
LRRK2 does not directly phosphorylate Thr^175^ tau *in vitro*. **(A)** Western blot analysis of the relative level of pThr^175^ tau (normalized to total tau) in HEK293T cells co-transfected with 2N4R WT-tau and either LRRK2-WT, LRRK2-G2019S (constitutively active) or LRRK2-3XKD (inactive). There was no difference in the level of pThr^175^ tau between cells transfected with LRRK2-WT, LRRK2-G2019S and LRRK2-3XKD *p* > 0.05. **(B)** Western blot analysis of the relative level of pThr^231^ tau (normalized to total tau) in HEK293T cells co-transfected with 2N4R WT-tau and either LRRK2-WT, LRRK2-G2019S or LRRK2-3XKD. LRRK2 did not increase the level of pThr^231^ tau *p* > 0.05. **(C)** Western blot analysis of the relative level of pSer^9^-GSK3β (normalized to total GSK3β) in HEK293T cells co-transfected with 2N4R WT-tau and either LRRK2-WT, LRRK2-G2019S or LRRK2-3XKD. The level of pSer^9^-GSK3β normalized to total GSK3β did not differ between cells expressing LRRK2-G2019S (active) or LRRK2-WT and LRRK2-3XKD (inactive) *p* > 0.05. **(D)** Western blot analysis of the relative level of pRab10 in HEK293T cells co-transfected with Rab10 and either LRRK2-WT, LRRK2-G2019S or LRRK2-3XKD (expression represented in slot blot). pRab10 was increased in cells expressing LRRK2-G2019S compared to LRRK2-WT (*p* = 0.0038) and LRRK2-3XKD (*p* = 0.0016), indicating that LRRK2-G2019S is active and capable of acting on substrates. A One-way ANOVA followed by Tukey’s post-hoc test was conducted. ^**^p < 0.005.

Given that LRRK2 expression is increased within the first 10 days post-injury in TBI rodents, we investigated whether LRRK2 could phosphorylate tau at Thr^175^ *in vitro*. HEK293T cells were co-transfected with eGFP tagged 2N4R human WT-tau and one of three LRRK2 constructs: LRRK2-WT, LRRK2-G2019S (constitutively active) or LRRK2-3XKD (inactive) at 1, 2 and 4 μg. Transfection of WT-tau alone was used as a control. First, we measured the level of LRRK2 expression (Supplementary Figure S1). Given that each LRRK2 construct expressed at differing levels, when measuring pTau and pGSK3β we compared conditions with the most similar level of LRRK2 expression. There was no significant difference in the level of pThr^175^ tau in cells overexpressing the constitutively active LRRK2-G2019S compared to LRRK2-WT or the kinase-dead LRRK2-3XKD (Figure 3A; Supplementary Figure S2B). To confirm these data, we co-transfected WT tau and a second kinase dead LRRK2 mutant, LRRK2-K1609M (Gloeckner et al., 2006; Chia et al., 2014). There was no significant difference in the level of pThr^175^ tau between LRRK2-G2019S or LRRK2-WT and LRRK2-K1906M (data not shown). Taken together, these results suggest that LRRK2 is not involved in the phosphorylation of Thr^175^ of tau.

To confirm our findings, we examined downstream markers of our previously characterized cellular pathway, including pSer^9^-GSK3β (as a marker of GSK3β activity) and pThr^231^ tau. If LRRK2 is involved in Thr^175^ tau phosphorylation, these downstream markers should also be increased. We observed no significant difference in pThr^231^ tau (Figure 3B) or pSer^9^-GSK3β (Figure 3C) between LRRK2-G2019S and LRRK2-WT or LRRK2-3XKD expressing cells. These data show that LRRK2 does not initiate the cellular cascade associated with pThr^175^ tau-induced fibril formation, further supporting that LRRK2 does not phosphorylate Thr^175^ of tau.

To validate that the constructs were active, we examined the phosphorylation of a known LRRK2 substrate, Rab10 (Steger et al., 2016). HEK293T cells were co-transfected with eGFP-tagged Rab10 and one of three LRRK2 constructs. The level of pRab10 was significantly greater with the expression of LRRK2-G2019S compared to LRRK2-WT (*p* = 0.0038) or LRRK2-3XKD (*p* = 0.0016) (Figure 3D), showing that the findings from the *in vitro* experiments were not due to inactivity of LRRK2.

## 4. Discussion

The phosphorylation of tau at Thr^175^ can initiate a cellular cascade resulting in PAD exposure, GSK3β activation, Thr^231^ tau phosphorylation and pathological tau fibril formation with subsequent cell death (Moszczynski et al., 2015; Hintermayer et al., 2019). We have previously shown that pThr^175^ tau is a common phospho-epitope amongst various neurodegenerative diseases and can be initiated by TBI (Moszczynski et al., 2017, 2018). However, it is currently unknown which kinase phosphorylates tau at Thr^175^. Determining which kinase is responsible for initiating this pathological cellular cascade is essential to understanding the pathogenicity of tauopathies and TBI. Additionally, targeting the pThr^175^ tau pathway through kinase inhibition may be a potential therapeutic strategy to reduce tau phosphorylation and aggregation. In this study, we investigated the potential role of LRRK2 in the phosphorylation of Thr^175^ of tau in a rodent model of TBI and *in vitro*. First, we found that LRRK2 protein expression is increased in infiltrating microglia and astrocytes and is mislocalized to the neurites in the first 10 days following a single TBI in rodents, which temporally and spatially aligns with the expression of pThr^175^ tau. However, overexpression of a constitutively active mutant, LRRK2-G2019S, did not induce the phosphorylation of Thr^175^ tau in HEK293T cells, nor did it elicit the expression of other downstream events (pSer^9^-GSK3β and pThr^231^ tau) although it did increase phosphorylation of Rab10, a known LRRK2 substrate. These results show that LRRK2 is rapidly upregulated following TBI but does not contribute to the induction of pThr^175^ tau or activate the cascade leading to fibril formation elicited by pThr^175^ of tau.

LRRK2 has predominantly been investigated in PD where familial mutations contribute to disease pathogenesis by mediating various neurodegenerative-associated pathways (Cookson, 2015). However, its role in other neurodegenerative diseases is less well understood. We show in a rodent model of experimental TBI that there is an upregulation in total LRRK2 over the first 10 days post-injury compared to non-injured controls. This is consistent with previous studies showing a robust increase in LRRK2 protein and mRNA expression in CCI and weight-drop models of TBI in rodents (Bae et al., 2018; Rui et al., 2018). Similar to our findings, the increase in total LRRK2 was predominantly located in the peri-contusion region, suggesting that it may play a role in responding to the physical cortical and axonal injury sustained. Over the first 10 days, LRRK2 was also increased in the hippocampus.

While previous studies have shown increased LRRK2 in neurons (Greggio et al., 2008; Bae et al., 2018), the upregulation in microglia and astrocytes in TBI is less understood. Given that microglia and astrocytes play an essential role in the acute and chronic sequelae that occur post-TBI, our observation of increased LRRK2 expression in glial cells may suggest a role in the neuroinflammatory response. Consistent with this, following TBI, increased LRRK2 expression has been observed to be associated with increased hypoxia-inducible factor 1-α (HIF-1α), while inhibition of LRRK2 reduced neurodegeneration, neuroinflammation and cognitive deficits (Bae et al., 2018). LRRK2 can also indirectly activate the mitogen-activated protein kinase (MAPK) family (Gloeckner et al., 2009; Chen et al., 2012; Yoon et al., 2017), which plays a critical role in the pathophysiology of TBI.

Despite the association between LRRK2, TBI and tau phosphorylation, we showed that overexpression of a constitutively active LRRK2 variant (LRRK2-G2019S) did not significantly increase the level of pThr175 tau compared to overexpression of LRRK2-WT and LRRK2-3XKD. This is in contrast to a previous study that showed that LRRK2-G2019S increased tau phosphorylation at multiple sites, including Thr^175^. The difference in findings may be attributed to the nature in which the role of LRRK2 to phosphorylate Thr^175^ tau was investigated. Our study used overexpression of LRRK2 in cell culture, whereas the previous study by Bailey et al. (2013) was performed by incubating purified LRRK2 and tau in a kinase assay *in vitro*. We utilized the 2N4R human tau isoform because we previously showed that pseudophosphorylation of Thr^175^ of tau initiated fibril formation regardless of isoform (Gohar et al., 2009), while the previous study used the 0N3R human tau isoform. We have previously demonstrated that multiple isoforms of tau are phosphorylated at Thr^175^ in human ALS and CTE tissue (Moszczynski et al., 2018). Furthermore, the results from Bailey et al. (2013) found that Thr^175^ tau phosphorylation occurred at a rate of less than 10%, suggesting this is a rare phenomenon. Additionally, incubation of LRRK2-G2019S with a short tau fragment containing Thr^175^ did not result in predominant phosphorylation (Bailey et al., 2013). While we cannot rule out that LRRK2 may play a minor role in the phosphorylation of Thr^175^ at a level that we could not measure using our techniques, we showed that there is no significant difference in the level of pThr^175^ tau or downstream markers elicited from this phosphorylation of tau. Our finding is supported by multiple studies that have failed to identify a tangible link between LRRK2 and tau phosphorylation (Mikhail et al., 2015; Henderson et al., 2021).

In conclusion, we have demonstrated that LRRK2 protein expression is increased acutely following experimental TBI and is sustained for 10 days post-injury. The spatial and temporal increase in LRRK2 coincides with the phosphorylation of Thr^175^ tau, a critical phospho-epitope in the pathogenesis of tauopathies. However, we show that LRRK2 does not directly phosphorylate tau at Thr^175^ *in vitro*.

## Data availability statement

The raw data supporting the conclusions of this article will be made available by the authors, without undue reservation.

## Ethics statement

The animal study was approved by Western University Animal Care Committee (AUP #2017-135). The study was conducted in accordance with the local legislation and institutional requirements.

## Author contributions

ND: Conceptualization, Data curation, Formal analysis, Investigation, Methodology, Writing – original draft. MH: Methodology. MaS: Methodology. ES: Methodology. KV: Conceptualization, Formal analysis, Methodology, Supervision, Writing – review & editing. MiS: Conceptualization, Funding acquisition, Project administration, Supervision, Writing – review & editing.
