# Supplementary material for: Upregulation of LRRK2 following traumatic brain injury does not directly phosphorylate Thr175 tau
Source: Front Cell Neurosci. 2023 Nov 8;17:1272899. doi: 10.3389/fncel.2023.1272899 (PMC10663351; doi:10.3389/fncel.2023.1272899)
Supplement: Supplementary file 1 [file Image_1.pdf]

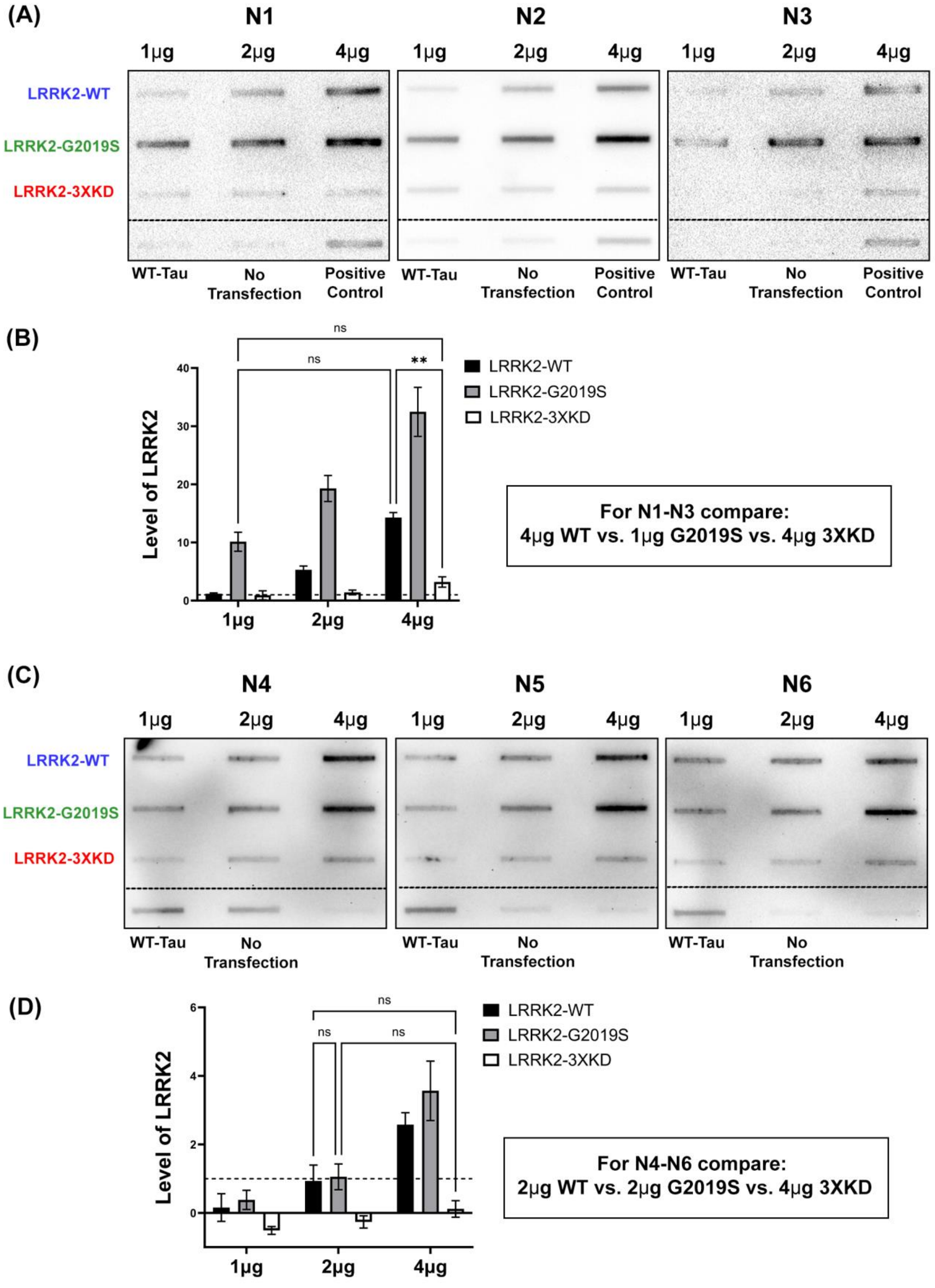
(A)
N1
N2
N3
1µg
2µg
4µg
LRRK2-WT
LRRK2-G2019S
LRRK2-3XKD
WT-Tau
No Transfection
Positive Control
(B)
ns
ns
**
Level of LRRK2
40
30
20
10
0
1µg
2µg
4µg
LRRK2-WT
LRRK2-G2019S
LRRK2-3XKD
For N1-N3 compare:
4µg WT vs. 1µg G2019S vs. 4µg 3XKD
(C)
N4
N5
N6
1µg
2µg
4µg
LRRK2-WT
LRRK2-G2019S
LRRK2-3XKD
WT-Tau
No Transfection
(D)
ns
ns
ns
Level of LRRK2
6
4
2
0
1µg
2µg
4µg
LRRK2-WT
LRRK2-G2019S
LRRK2-3XKD
For N4-N6 compare:
2µg WT vs. 2µg G2019S vs. 4µg 3XKD

Supplemental Figure 1. *In vitro* LRRK2 expression. (A) Slot blots for total LRRK2 expression in HEK293T cells co-transfected with WT-Tau and either 1, 2 or 4µg of LRRK2-WT, LRRK2-G2019S or LRRK2-3XKD DNA for replicates N1-3. (B) Densiometric analysis of slot blots for the relative level of LRRK2 expression in replicates N1-3. In order to ensure that the construct-specific variability in LRRK2 expression was accounted for, all immunoblot analysis for N1-3 was conducted using 4µg LRRK2-WT, 1µg LRRK2-G2019S and 4µg LRRK2-3XKD DNA. (C) Slot blots for total LRRK2 expression in HEK293T cells co-transfected with WT-Tau and either 1, 2 or 4µg of LRRK2-WT, LRRK2-G2019S or LRRK2-3XKD DNA, for replicates N4-6. (D) Densiometric analysis of slot blots for the relative level of LRRK2 expression in replicates N4-6. Given construct-specific differences in LRRK2 expression, as with experiments N1-3 we controlled for these differences by quantifying $pThr^{175}$ tau using for N4-6 using 2µg LRRK2-WT, 2µg LRRK2-G2019S or 4µg LRRK2-3XKD DNA. A two-way ANOVA followed by Tukey’s post-hoc test was conducted. $^{**}p < 0.005$.
