# Supplementary material for: Upregulation of LRRK2 following traumatic brain injury does not directly phosphorylate Thr175 tau
Source: Front Cell Neurosci. 2023 Nov 8;17:1272899. doi: 10.3389/fncel.2023.1272899 (PMC10663351; doi:10.3389/fncel.2023.1272899)
Supplement: Supplementary file 2 [file Image_2.pdf]

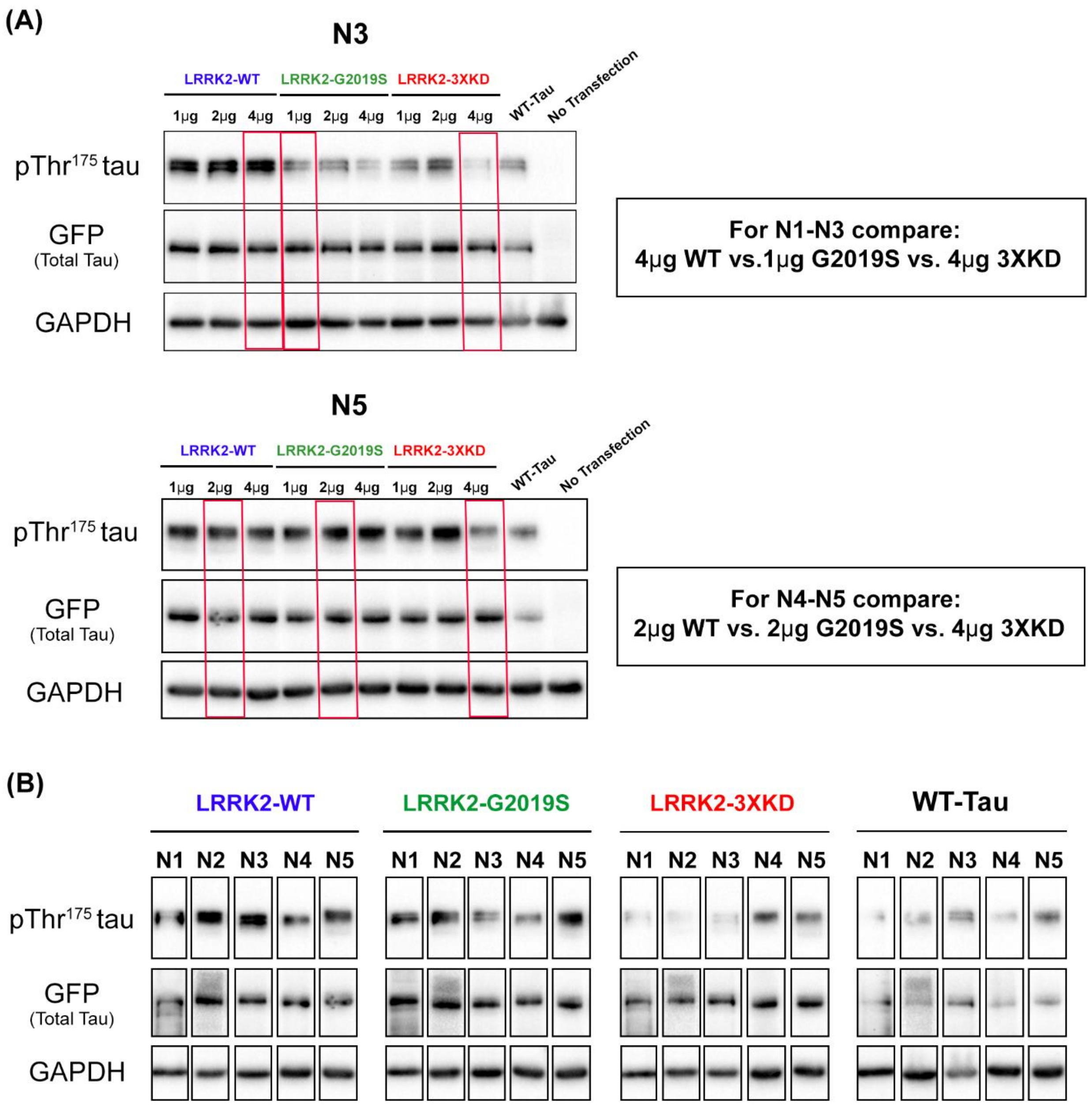

Supplemental Figure 2. *In vitro* level of pThr^{175} tau. (A) Representative original Western blots probing pThr^{175} tau, GFP (total tau) and GAPDH, used for densiometric analysis. For experiment N3, 4µg LRRK2-WT, 1µg LRRK2-G2019S, and 4µg LRRK2-3XKD DNA conditions were used for analysis to account for construct-specific variances in LRRK2 expression whereas for N5, 2µg LRRK2-WT, 2µg LRRK2-G2019S and 4µg LRRK2-3XKD DNA conditions were used. (B) Composite image of raw Western blots used for densiometric analysis. For N1-3, 4µg LRRK2-WT, 1µg LRRK2-G2019S and 4µg LRRK2-3XKD DNA was analyzed whereas for N4-5, 2µg LRRK2-WT, 2µg LRRK2-G2019S and 4µg LRRK2-3XKD DNA was analyzed.
